# Case Report: Epidemiology, clinical features and progress in disease management for lymphoepithelial carcinoma of salivary glands: report of 3 cases

**DOI:** 10.3389/fonc.2026.1863285

**Published:** 2026-07-17

**Authors:** Shunyi Huang, Cenwen Liu, Xueying Zeng, Zihan Huang, Feng Lin, Yongyuan Chen, Guanxiang Zhuo, Jiahui Lin, Dawei Lin, Qinqin Chen, Xinxin Chen, Yunyun Mei, Qing Guan

**Affiliations:** 1 Department of Head & Neck Surgery, Xiamen Cancer Hospital, Xiamen, China; 2 Department of Pathology, Xiamen Cancer Hospital, Xiamen, China; 3 Xinjiang Medical University, Urumqi, China; 4 Department of General Surgery, The First Hospital of Putian City, Putian, China; 5 Xiamen Cancer Hospital, Xiamen, China; 6 Department of Head and Neck Surgery, Fudan University Shanghai Cancer Center, Shanghai, China; 7 Department of Oncology, Shanghai Medical College, Fudan University, Shanghai, China

**Keywords:** case report, combination therapy, Epstein-barr virus, head and neck cancer, lymphoepithelial carcinoma of salivary gland

## Abstract

**Background:**

Lymphoepithelial carcinoma is an extremely rare malignant tumor in salivary glands, so it is of great significance to understand and summarize the disease characteristics and treatment experience.

**Methods:**

We reported 3 lymphoepithelial carcinoma cases undergoing surgical treatment and postoperative radiotherapy.

**Results:**

All cases were pathologically diagnosed with lymphoepithelial carcinoma of salivary gland after surgery, among which two had cervical lymph node metastasis. 2 cases have been followed up for 15 months without local recurrence or distant metastasis.1 cases has been followed up for 15 months,hepatic metastasis was diagnosed at the 8th month after postoperative radiotherapy, and received surgery for the liver lesion.

**Conclusion:**

Lymphoepithelial carcinoma of salivary gland has complex etiology and still needs a combined treatment based on surgery. Targeted therapy and immunotherapy may be used as new treatments in the future.

## 1 Introduction

As a type of head and neck cancer, lymphoepithelial carcinoma (LEC) is an extremely rare malignant tumor in salivary glands,accounts for approximately 0.4% of malignant salivary gland tumors,among which 80% of cases arise primarily from the parotid gland (1).LEC was first reported by Hilderman in 1962 (2). As an undifferentiated or poorly differentiated squamous cell carcinoma, Epstein-Barr virus (EBV) plays an important etiological role in the development of LEC (3, 4), while its pathogenesis, diagnosis and treatment strategies have been the focus of clinical research. Early salivary gland lymphoepithelial carcinoma typically presents as a slowly growing painless mass in the parotid or submandibular area, while pain may occur in partial cases. Facial nerve invasion results in ipsilateral facial palsy featuring mouth askew, difficulty closing eyes and impaired facial movement (5). Preoperative ultrasound, contrast-enhanced CT and MRI assess tumor features, location and peripheral tissue invasion (6). Early PET-CT detects lymph node and distant metastasis,may helps to guide treatment planning (7). Preoperative fine-needle aspiration cytology (FNAC), a major needle biopsy method, confirms pathological diagnosis. Surgical resection is the first-line basic treatment for early lesions. Locally advanced or metastatic patients receive individualized combined treatments such as radiotherapy, chemotherapy, immunotherapy and targeted therapy.Combination therapy based on surgery and radiotherapy remains the current clinical treatment, but not all patients can obtain benefit, so accurate diagnosis and effective treatment are urgently needed to improve the survival rate. In this study, we report 3 patients with LEC of salivary gland receiving surgical treatment.

## 2 Case presentation

**Figure 1:**
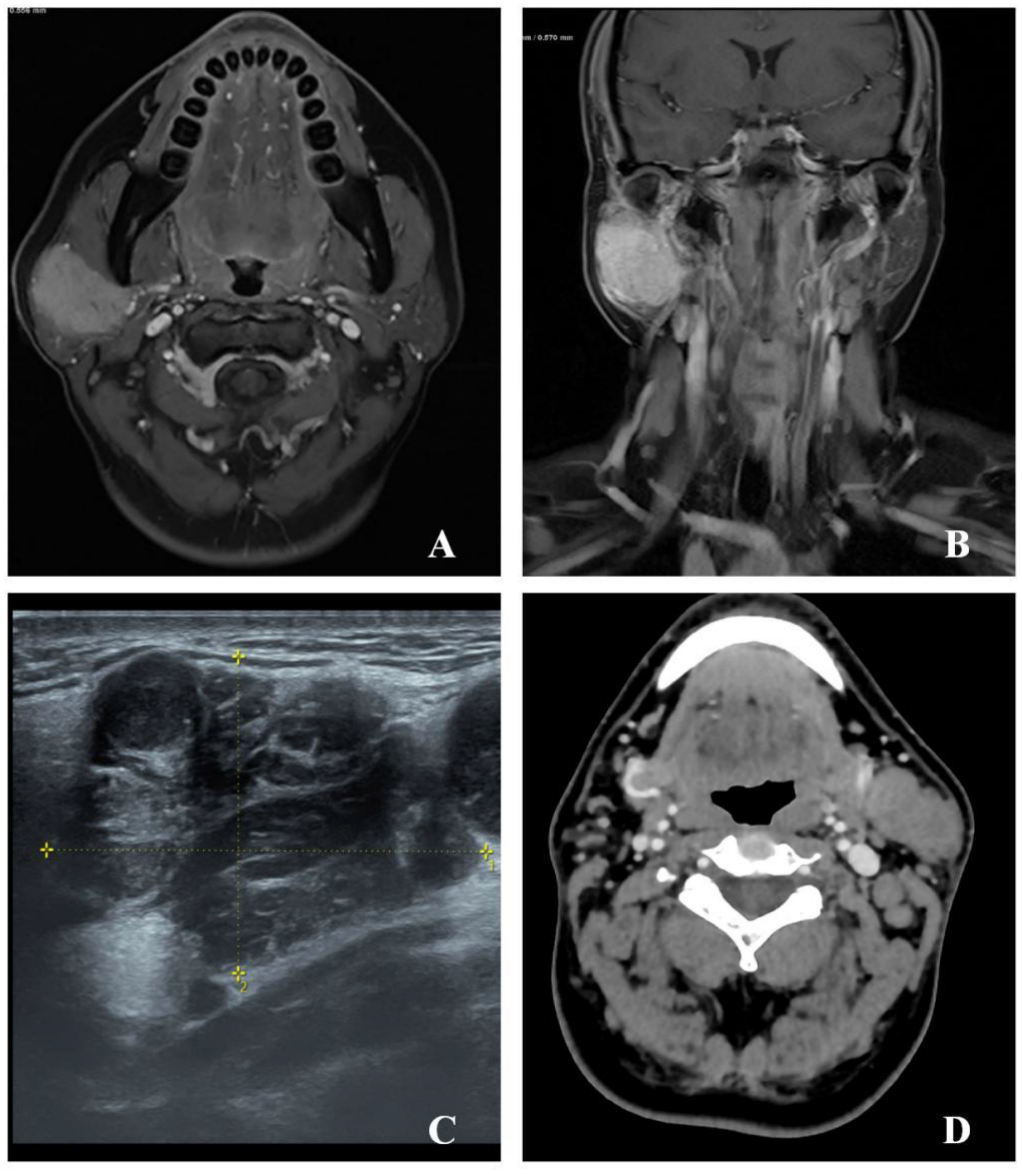
Lymphoepithelial carcinoma of salivary glands. MRI, Axial contrast-enhanced T1-weighted imaging **(A)** and coronal contrast-enhanced T1-weighted imaging **(B)** demonstrated a mass with relatively clear boundaries and heterogeneous mild-to-moderate enhancement. Color Doppler, A hypoechoic mass was detected in the lower pole of the left parotid gland, featuring clear boundaries, an irregular and lobulated shape, a relatively dense internal structure, and linear hyperechoic foci **(C)**. CT, An irregularly shaped mass with heterogeneous enhancement was observed in the left parotid gland region **(D)**.

Case 1: A 41-year-old female patient was admitted to our hospital due to right parotid gland mass for 1 week. Physical examination: A palpable mass with 2.5*2.0 cm in size was in the lower pole of right parotid gland, with moderate texture and clear boundary, general mobility and no obvious tenderness. No significantly enlarged lymph nodes were found in bilateral neck and supraclavicular region.(The normal size range of lymph node is 0.2-0.5cm.)Color Doppler imaging: A 3.3*2.4 cm solid nodule in right parotid gland, absence of right thyroid gland, no significant occupying lesion in left thyroid gland, bilateral neck and supraclavicular region. Magnetic resonance imaging (MRI): Oval abnormal signal shadows were observed in right parotid region, ill-defined boundary with 3.0*2.4 cm, slightly high signal intensity on T2-weighted imaging (T2WI), equal signal on T1-weighted imaging (T1WI), significant high signal intensity on diffusion-weighted imaging (DWI), significant low signal intensity on apparent diffusion coefficient (ADC). Less homogeneous signal intensity, heterogeneous mild to moderate enhancement after contrast enhancement, clear boundary, several slightly larger lymph node shadows scattered in bilateral neck (**Figures 1A, B**). No significant abnormality in nasopharynx or oropharynx. FNAC biopsy report: (Solid nodule in right parotid gland) numerous lymphocytes, several small piles of heterocysts, suspected poorly differentiated carcinoma.

Case 2: A 37-year-old male patient was admitted to our hospital with the chief complaint of “discovering a space-occupying lesion in the left parotid gland for 3 months”. Physical examination: A mass measuring approximately 4.5*3.0 cm was palpable in the left parotid gland, with hard texture, poor mobility, and no obvious tenderness. Multiple enlarged lymph nodes were palpable in the left neck with hard texture, while no significantly enlarged lymph nodes were found in the right neck or bilateral supraclavicular regions. Color Doppler imaging: A 4.5*2.6 cm hypoechoic nodule was observed in the lower pole of the left parotid gland, with relatively clear boundaries, irregular shape, and lobulated edges (**Figure 1C**). Multiple hypoechoic lesions were detected in the left neck, with clear boundaries and plump morphology, and no lymphoid hilum structure was seen. No significant occupying lesions were found in the right parotid gland, bilateral submandibular glands, right neck, or bilateral supraclavicular regions. Computed tomography (CT): A mass in the left parotid gland region with irregular shape and heterogeneous enhancement, accompanied by multiple enhanced lymph nodes in the left neck (some enlarged) (**Figure 1D**). MRI: A mass in the left parotid gland region and multiple enlarged lymph nodes in the left neck, considering malignant tumor. FNAC biopsy report: (Left parotid gland mass) poorly differentiated carcinoma; parotid lymphoepithelial carcinoma is considered after excluding metastases such as nasopharyngeal non-keratinizing squamous cell carcinoma.

Case 3: A 26-year-old male patient was admitted to our hospital due to right cervical lymphadenopathy for 2 months. Physical examination: Several palpable confluent masses were in the right parotid gland, with the largest 4.0*3.0 cm in size, firm and fixed texture, ill-defined boundary, and no obvious tenderness. Multiple enlarged lymph nodes in right neck, among which the larger one was in V region with 2.0*2.0 cm in size, firm and fixed texture, ill-defined boundary, and no obvious tenderness. No enlarged lymph nodes in left neck. Color Doppler imaging: Multiple enlarged lymph nodes in Ib, II, III, IV and V regions of right neck, no enlarged lymph nodes in left neck. MRI: Patchy enhancement of the right parotid gland, multiple enlarged lymph nodes in the right neck and parotid region, which tended to metastasize, and multiple slightly larger lymph nodes in the left neck. FNAC biopsy report: (Right cervical lymph node) metastatic lymph node epithelial carcinoma. Laryngoscopy and nasopharyngoscopy revealed no significant abnormalities. Positron emission tomography/CT (PET/CT): Multiple nodules in right parotid gland, ill-defined boundary, with the largest 1.7*1.5 cm in size, increased fluorodeoxyglucose (FDG), malignant tumors were considered.

## 3 Results

All three patients directly received surgical treatment without pre-operative medications. Case 1 and case 2 underwent right radical parotidectomy + submandibular gland resection at the department of Head and Neck Surgery in Fudan University Shanghai Cancer Center, while case 3 at the Xiamen Branch, Fudan University Shanghai Cancer Center. Cervical lymph node metastasis may occur at an early stage in patients with salivary gland lymphoepithelial carcinoma. Ipsilateral cervical lymph node metastases are detected in 17%–30% of patients when diagnosed, presenting as hard, fixed cervical masses predominantly involving intraparotid, level II and level Ib lymph nodes. Previous studies have confirmed that occult cervical lymph node metastases are present in 20%–22% of patients (8). Neither preoperative imaging examination of Case 1 nor Case 2 indicated lymph node metastasis, both patients underwent elective neck dissection (levels I-III).Preoperative imaging revealed multiple cervical lymph node metastases in Case 3, who subsequently received radical neck dissection(levels I-V).

Postoperative pathology of case 1: (Right parotid gland) nests of atypical epithelial cells in highly proliferative lymphoid tissue, could not exclude possibility of LEC, tumor size 2.0*1.5 cm. (Right cervical lymph node) no metastasis in lymph nodes (0/14). Immunohistochemistry: AE1/AE3 (+), CK5/6 (+), P16 (partly +), CK7(-), CK20(-), CD117 (+), calponin (-), Ki-67 (around 70%+), P63 (+), EBV-encoded RNA(EBER) (+).

Postoperative pathology of case 2: (Left parotid gland and left submandibular gland) LEC. Tumor size: 4.0*2.5*2.0 cm. Vascular invasion (+), perineural invasion (+). Cancer metastases were detected in 3 out of 9 surrounding lymph nodes. (Left cervical lymph nodes) Cancer metastases were detected in 6 out of 27 lymph nodes. Immunohistochemistry: AE1/AE3 (+), P63 (+), P40 (+), EBER (+), EGFR (+), Ki-67 (approximately 70%+), CK5/6 (+), P16 (-), Syn (-), S-100 (-), Programmed death-ligand 1(PD-L1) 22C3 (CPS approximately 80%),GATA binding protein 3 (GATA3) (-), trichorhinophalangeal syndrome type 1 (TRPS1) (-).

**Figure 2:**
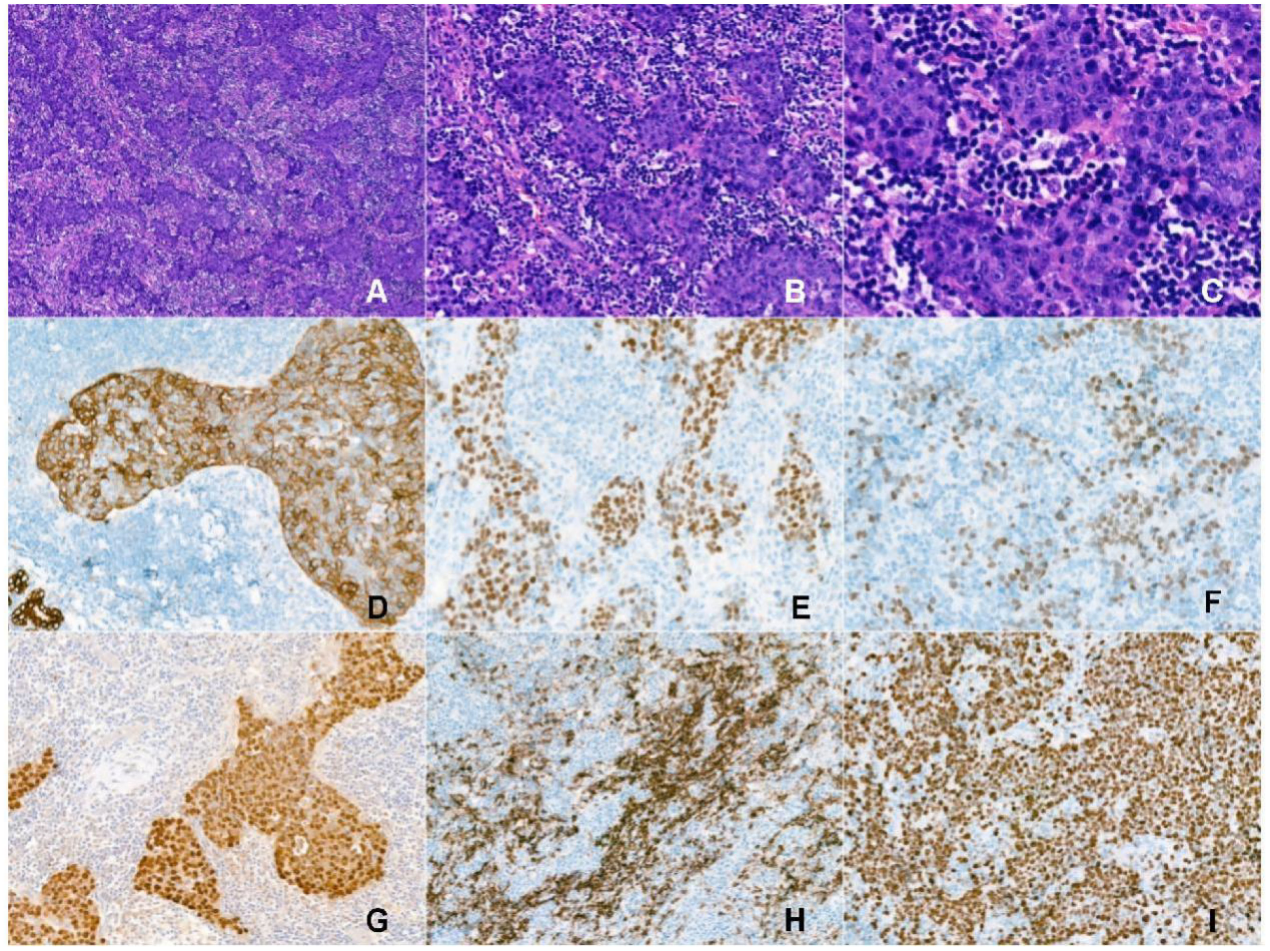
Microscopic morphology of lymphoepithelial carcinoma of salivary glands. **(A)** The tumor exhibits sheets, nests, and cord-like structures with infiltrative growth (Magnification: 40x). **(B)** The tumor cells are polygonal with indistinct cell borders and syncytial appearance, accompanied by abundant lymphocytic infiltration in the surrounding stroma (Magnification: 200x). **(C)** The tumor cells show abundant cytoplasm, vesicular nuclei, and prominent nucleoli (Magnification: 400x). **(D)** The tumor cells are positive for AE1/AE3 (Magnification: 200x). **(E)** The tumor cells are positive for P40 (Magnification: 200x). **(F)** Tumor cells are positive for P63 (Magnification: 200x). **(G)** Epstein-Barr Virus Encoded Small RNAs(EBER) *in situ* hybridization shows diffuse positivity in the tumor cells (Magnification: 200x). **(H)** The tumor cells are negative for CD20, while abundant surrounding lymphocytes are positive (Magnification: 100x). **(I)** The Ki-67 index is high, approximately 90% (Magnification: 200x).

Postoperative pathology of case 3: (Superficial lobe of right parotid gland) LEC, infiltrating the surrounding fibroadipose tissue. Tumor size: 62.52.5 cm. Vascular tumor thrombus (+), no definite perineural invasion. (Deep lobe of right parotid gland) Cancer involvement identified. (Right submandibular gland) No cancer involvement. (Right cervical lymph nodes) Cancer metastases detected in 9 out of 43 lymph nodes. Immunohistochemistry: AE1/AE3 (+), CK5/6 (+), P63 (+), P40 (+), EBER (+), SOX10 (-), D2-40 (vascular +), Ki-67 (90%+), CD20 (-), CD5 (-), P53 (partly +), Bcl-2 (+), CD117 (+) (**Figure 2**). Additional note: No positive human papillomavirus (HPV) subtypes detected.

All 3 cases received postoperative radiotherapy.

## 4 Discussion

This study investigated three LEC patients receiving surgical treatment at the department of Head and Neck Surgery in Fudan University Shanghai Cancer Center. By summarizing disease onset, clinical features, postoperative pathology, treatments and prognosis, we expect to provide basis for further studies on LEC.

### 4.1 Epidemiology and etiology

It has been confirmed that incidence of LEC present with significantly regional charateristics, with endemic areas mainly concentrated in Greenland, northern Canada, Alaska, Southeast Asia, Japan, and South China (9–12). In addition to geography, the disease showed a preference in terms of ethnicity. The endemic population is mainly Arctic Inuit, Southeast Asian, East Asian (Japan, southern China), and nearly half are Indigenous Arctic and Asian populations (13). While on the other hand, incidence rates of individuals of European descent, western countries (the United States, Britain and other European countries) and other non-epidemic areas are very low, with few relevant reports (10, 11, 14, 15). Besides, White individuals account for only about 7% of case reports in non-endemic areas. In the United States, the primary LEC population consists of older White adults with relatively good heath (9, 10). The age range of onset spans a wide range and is more common between 20 and 60 years, with a median age of about 40 and 62 years in endemic and non-endemic area, separately. Apart from the influence of ethnic factors, the mean age of patients in non-endemic areas was older than that in endemic areas (10, 15). Some literatures (10, 16, 17) support the lack of a significant difference in gender, while other studies (15, 18, 19) report inconsistent results with more female patients than male patients. Conversely, male patients predominate in studies of the Chinese population (15, 18, 19).

The pathogenesis is complex and is thought to result from the interaction of factors such as ethnicity (genetic), environment (geographic), behavior (smoking, alcohol consumption), and viruses, while EBV infection is a key element for occurrence of LEC (9, 13, 20). It is worth mentioning that acquired immunodeficiencysyndrome(AIDS) patients have an increased risk of LEC, suggesting that immunodeficiency and immunosuppression may play a role in the etiology (21).

### 4.2 Pathological features and classification

Pathology remains the gold standard for diagnosis of LEC of salivary gland, which can be described as a lobulated, brownish-white firm mass with mostly ill-defined borders and no obvious capsule, most of which are concentrated between 1 and 10 cm in diameter and up to about 50–70 cm in larger ones (22, 23). Tumor cells of LEC are characterized by undifferentiated epithelial cells arranged in cords, nests, sheets, islands, or lobes, tumor cells are spindle-shaped or polygonal, occasionally round or oval, nuclei are vesicular, basophilic, and prominent eosinophilic nucleoli, one or more. Tumor cells sometimes have blurred nuclei, and their pleomorphism is often easily recognized. Foci of squamous differentiation including keratinization can be demonstrated and identified in tumor cells. The syncytia of tumor islands have a high nucleocytoplasmic ratio, the peripheral lymphoid stroma is eosinophilic, delicate and sparse, and not deeply stained, and is mainly composed of polyclonal B lymphocytes, T lymphocytes, and plasma cells, that is, separated or infiltrated by lymphoid stroma composed of small lymphocytes and plasma cells (22, 24). Germinal centers can be present in lymphoid stroma, and mitotic and apoptotic bodies have also been shown in studies (24, 25). A large number of reactive histiocytes engulfed nuclear debris giving rise to a “stellate” appearance that is not characteristic of benign lymphoepithelial lesions of salivary glands (22, 24). Peripheral nerve invasion or infiltration is currently an important finding (22, 24). The degree of peripheral lymphocyte infiltration and the number of tumor epithelial cell types have an impact on the cytological morphology of LEC (26). It should be noted that other high-grade salivary gland tumors cytologically overlap with LEC, so cytological features are sometimes not unique to LEC. For example, high-grade mucoepidermoid carcinoma has high-grade poorly differentiated cells and lacks mucinous cells close to LEC (13).

Histological type of salivary gland LEC is based on morphological characteristics and differentiation of tumor cells. LEC is usually composed of squamous cells, intermediate cells, and mucus-secreting cells, which are present in different proportions in different tumors (22). According to the histological appearance of tumor, frequency of cell division, cellular atypia, and degree of necrosis, LEC can be classified to low-grade or high-grade (22). Low-grade LEC manifests as poor differentiation, less cellular atypia, low frequency of cell division, while high-grade LEC presents with good differentiation, more cellular atypia, high frequency of cell division, and often accompanied by necrosis (23). In addition, grading can also serve as auxiliary diagnosis through specific gene mutations and molecular markers, such as Mastermind Like Transcriptional Coactivator 2 MAML2 gene rearrangement who is more common in low-grade and intermediate-grade salivary gland LEC (24).

### 4.3 Immunohistochemical features

Immunohistochemical features of salivary gland LEC often showed positive pan-cytokeratin, CK5/6, epithelial membrane antigen (EMA), p40 and p63 (22, 23, 27), which help to distinguish them from other types of salivary gland tumors (26). AE1/AE3, CAM5.2 and CK903 also have reactivity (23). CD117 may respond in some patients, but p16 is negative, which is associated with the presence of latent membrane protein 1 (LMP-1) (17, 22, 23). Mucus-related markers, such as MUC1 and MUC4, are also frequently expressed and help to confirm the presence of mucus-secreting cells (23). Ki-67 proliferation index is usually high in high-grade LEC, reflecting the high proliferative activity of tumor cells (23). Besides, immunohistochemical features of LEC include overexpression of Epidermal Growth Factor Receptor EGFR, which is related to tumor aggressiveness and poor prognosis in some cases (23). *In situ* hybridization of EBER is an important indicator of LEC, and positivity can be detected in endemic areas, with the opposite probability in non-endemic areas (14, 18, 22, 23). Immunohistochemistry can be used to distinguish from other epithelial malignancies, such as non-pigmented melanoma detected by cytokeratin, S-100 protein and HMB45 immunostaining, and common leukocyte antigen(CLA) staining to distinguish large cell or anaplastic lymphoma (19). However, it is difficult to differentiate LEC from nasopharyngeal carcinoma (NPC) because of the overlapping histopathological, immunohistochemical, and cytogenetic features (19, 22). CD3 and CD20 have some value in studying immunohistochemistry (19, 22), but more experiments are required. Somatostatin receptor 2 (SSTR2) expression is present in some patients with lymphoepithelial carcinoma of the parotid gland, which may be helpful for guiding relevant targeted therapies in the future (26). It is worth mentioning that mutually exclusive expression of p16 and Human epidermal growth factor receptor 2 (HER2) has been reported in patients with EBV-associated salivary gland LEC, which poses some diagnostic challenges for some tumors with atypical immunophenotypes (23).

### 4.4 Molecular pathology

In recent years, significant development is obtained in molecular pathology of salivary gland LEC. One important molecular feature is CRTC1/3-MAML2 gene fusion which can be detected in approximately 38-82% of cases (19). Such gene fusion always contributes to better prognosis, especially more common in low-grade and intermediate-grade LEC (19). In addition, EGFR gene amplification and overexpression have also been reported in some cases, pointing out a potential role for anti-EGFR therapy (19). Other molecular markers such as CLDN2, CLDN7 and CLDN18 have also been reported, and these markers may provide new ideas for future targeted therapies (19). Overall, advances in molecular pathology have not only contributed to the diagnosis and classification of salivary gland LEC, but also provided new possibilities for individualized treatment.

### 4.5 Imaging features

For patients who present with a head and neck mass, routine available examinations include head and neck palpation, color Doppler ultrasound, CT, and MR examinations. Because of the low clinical incidence, current literature on imaging features of primary salivary gland LEC is limited and relevant summaries are limited (7, 28). To investigate the analysis of imaging features can choose CT or MR examination. LEC can be found more frequently involving the parotid gland, followed by the submandibular gland, and relatively few other salivary glands in imaging studies (7)^2^ (29), with main imaging features as follows: (1) most salivary gland LEC present as single or multiple intra-glandular masses, solid, less regular in shape, uniform in density, lobulated nodules, ill-defined borders, can be complicated by calcification or cystic degeneration; (2) most lesions show moderate or significant homogeneous enhancing masses on CT or MR; (3) most lesions lack necrosis, and no enhancement of necrotic parts or regions; (4) possible cervical lymph node metastasis during the examination, and intra-glandular lymph node metastasis may be observed in some parotid cases (7, 29). In the process of imaging examination, attention should be paid that are easily confused with benign tumors: (1) the pseudocapsule formed by malignant tumors promotes edge of the lesion getting clear which is easily mistaken for benign lesions (7); (2) multiple epithelial malignant lesions in the parotid gland may be highly similar to multiple Warthin tumors in the parotid gland in CT and MR image findings and should be differentiated; (3) solitary salivary gland LEC may or may not be associated with regional lymph node metastasis, and the characteristics are insufficient and sometimes difficult to differentiate from some oral and maxillofacial benign tumors (29). Since imaging examination is non-specific, as a link in clinical examination, should be combined with puncture pathology and immunohistochemistry and other examination results to confirm the diagnosis and avoid misdiagnosis.

### 4.6 Clinical features and differential diagnosis

Salivary gland carcinoma has multiple subtypes, among which LEC is rare, accounting for only 0.4% of all salivary gland carcinoma (9, 20, 25). In addition to the name salivary gland LEC, lymphoepithelioid carcinoma, undifferentiated lymphoid stromal carcinoma, malignant lymphoepithelial lesions, and lymphoepithelial carcinoma can also be used to describe this disease (20, 22, 23). The most commonly affected site in LEC cases is the parotid gland, followed by the submandibular gland, while the sublingual and other minor salivary glands are relatively rare (20, 22, 23). The clinical presentation is not specific and sometimes difficult to characterize by a simple clinical presentation, especially preoperatively. The more common symptoms in patients are salivary gland enlargement or palpable masses in the salivary gland region with clear or unclear borders, regular or irregular shape, generally not accompanied by pain or facial paralysis, and the duration of symptoms is not fixed, ranging from days to months, or even years (9, 10, 22, 24). Some patients had significant perception of tumor progression, including an increase in weight or diameter of the mass (9, 24). In advanced cases, the tumor can be observed fixed in the underlying tissue and can invade the surrounding soft tissue or overlying skin, with or without ulcerated foci (7, 28). Metastases to local or cervical lymph nodes are often detected during the examination, but they do not necessarily present as enlargement of cervical lymph nodes (20, 22, 23). LEC of salivary gland is usually differentiated from benign epithelial lesions, malignant lymphoma of head and neck, malignant melanoma, and metastatic carcinoma of other sites. Benign lymphoepithelial lesions such as lymphoepithelial sialadenitis lack morphologic evidence of malignancy and are predominantly benign components with no destructive infiltrates (13, 20, 22, 24). Negative immunohistochemical markers CD20 and CD45 can be used to differentiate from lymphoma, with cellular atypia appearing in epithelial cells rather than lymphocytes, and differences between the two can also be found at low magnification (9, 24). Metastatic carcinoma at other sites, particularly non-keratinizing undifferentiated nasopharyngeal carcinoma, should be carefully and carefully differentiated. Metastatic poorly differentiated squamous cell carcinoma can be diagnosed by intercellular bridges and keratinocyte eosinophilic cytoplasm (30). Malignant lymphoepithelial cells of the parotid gland overlap and are identical to undifferentiated nasopharyngeal carcinoma epithelium in cellular tissue and immunohistochemistry, and both may be EBV-positive, so collection of previous relevant medical history, upper respiratory endoscopy, or nasopharyngeal biopsy is essential to rule out primary tumor metastasis in the nasopharynx (9, 10, 22, 24). HPV-associated lymphoepithelial forms of oropharyngeal squamous cell carcinoma can also metastasize, with strong, diffuse, nuclear, and cytoplasmic p16 responses in HPV-positive or more than 70% of tumor cells and/or high-risk human papillomavirus ISH, which can be used to suggest that the primary tumor is located in the oropharynx (13, 20, 22, 24). Large cell undifferentiated carcinomas have strong mitoses, frequent cell necrosis, lack EBV and HPV markers, and lack histomorphologic features of glandular or epidermoid differentiation, which are helpful for differential diagnosis (13, 20, 22, 24). Furthermore, multiple reactive and neoplastic lesions such as reactive tumor-associated lymphoid hyperplasia and Warthin ‘s tumor, the diagnosis points inclued slow clinical onset, no atypia and destruction, epithelium maintains lobular shape, neoplastic epithelial cell changes, or have papillary structures (9, 24). Previous study (29) has indicated that prognosis of LEC may be related to age, race, marital status, tumor invasion, lymph node metastasis, and surgical treatment. Specifically, aged over 60 years and lymph node metastasis are associated with poor prognosis, and widowed or divorced patients tend to have better prognosis than unmarried or married patients.

### 4.7 Clinical treatments

In view of the rarity of salivary gland LEC, our study mainly discusses the current treatments including surgery, radiotherapy, chemotherapy, targeted therapy, and immunotherapy.

#### 4.7.1 Surgical treatment

According to the National Comprehensive Cancer Network (NCCN) guidelines (31), surgery is considered to be the most effective treatment of salivary gland LEC, requiring clear surgical margins and complete resection of the tumor lesion. Wider extent of resection is not significantly helpful for the treatment and prognosis, due to reduced progression-free survival and cervical fibrosis after radical neck dissection (9, 24). LEC arising in the parotid gland can be treated with superficial parotidectomy if it is confined to the superficial lobe, but total parotidectomy is more appropriate if the lesion is in the deep lobe or has reached locally advanced stage (9, 24). Cervical lymph node involvement is often observed in clinical cases, and lymph node metastasis is the development trend of the disease. Intraglandular lymph node metastasis or peripheral lymph node metastasis is most likely to occur, followed by supracervical lymph nodes, which spread sequentially to various lymphatic drainage areas of the neck, and supraclavicular lymph nodes can also be involved (9, 24). Clinically, treatment options for patients with negative cervical lymph nodes remain controversial, observation, prophylactic radiotherapy, or selective neck dissection can be the options (9)^1^ (24). Neck dissection is the treatment of cervical lymph node positivity, especially biopsy confirmed or imaging suspicious findings, it is recommended to complete therapeutic neck dissection at the time of initial surgery, followed by radiotherapy (9, 24). Surgical complications include impaired facial nerve function, facial depression deformity, and residual tumors, which have an impact on both quality of life and mental health of patients (32, 33).In our study,the tumor were assessed as resectable without surgical contraindications in all cases, they underwent surgery including primary tumor resection and neck dissection directly. In addition, some patients develop distant metastasis, which is common in the lung, bone, liver, brain and mediastinum. Local recurrence and distant metastasis easily lead to treatment failure. Therefore, it is necessary to carefully screen distant metastasis before selecting surgical treatment.

#### 4.7.2 Radiotherapy and chemotherapy

Salivary gland LEC is considered to be highly radiosensitive in a variety of salivary gland cancers, with a high rate of local tumor control and a significant effect in removing residual minimal disease lesions, so radiotherapy is recommended for clinical treatment (9, 24). Because tumors invade the skull base or protect nerves, surgery is usually difficult to completely remove the tumor, so surgery combined with postoperative radiotherapy is an appropriate treatment with satisfactory regional control (9, 24). Compared with surgery alone, postoperative radiotherapy can reduce the risk of recurrence, while not significantly increase the incidence of advanced cervical fibrosis toxicity, and improve progression-free survival and overall survival as well. Radical surgery combined with radiotherapy has been proved to have some effect in local tumor control, with a 5-year overall survival rate of about 50%-90% (33). Postoperative adjuvant radiotherapy should take the following high-risk factors into consideration: T3 or T4 tumors, margin closure or invasion, nerve or vascular invasion, lymph node involvement, high-grade histology, etc. When the maximum diameter of the primary lesion exceeds 3 cm and/or the number of positive lymph nodes exceeds 2, postoperative radiotherapy is regarded as an indispensable treatment (25, 34). A report suggests that elevated serum lactate dehydrogenase (LDH) before radiotherapy may be a predictor of distant metastasis after postoperative radiotherapy (33). For LEC residual disease that has been detected or suspected, radical radiotherapy with (or without) surgical treatment is effective and feasible, and if the condition persists after radiotherapy, the possibility of surgical treatment should still be preserved (33–36). It has been found that most radiotherapy treatments have mild toxicity, and acute reactions are common with oral mucositis and skin reactions, which may be due to lymph node drainage area involvement ipsilateral to the tumor (33). Chemotherapy, as palliative therapy, plays an important role in systemic therapy and is commonly used for the treatment of symptomatic local recurrence and/or metastatic disease that is not amenable to further surgery or radiotherapy (25, 37). Conventional chemotherapy regimens, such as cisplatin, 5-fluorouracil, or CAP (cisplatin, adriamycin, cyclophosphamide) are still the first-line treatment for patients with advanced disease, but after clinical testing, only a few such as cisplatin and 5-fluorouracil are considered effective (37). Another study has demonstrated that most patients with T1-T3 and N0-N2b disease do not experience significant additional benefit from adjuvant chemotherapy (19). Despite the histological similarity with NPC, chemotherapy regimens for salivary gland LEC requires more evidence to verify its clinical benefits (19). Postoperative chemoradiotherapy is more helpful in reducing tumor recurrence and distant metastasis rates than radiotherapy alone, with the disadvantage of a higher risk of mortality and drug toxicity (33)^1^ (34). Induction chemotherapy combined with concurrent chemoradiotherapy among advanced salivary gland is likely to be promoted in the future, but still lacking clinical validation with large sample size (33).

#### 4.7.3 Targeted therapy

Targeted therapy is now mostly used for the treatment of NPC, while very limited cases and experience in salivary gland LEC. Targeted agents such as imatinib, cetuximab, gefitinib, and trastuzumab are now being used to explore the treatment of salivary gland cancer, but still have no substantial breakthroughs (37). Targeted therapy is emerging and promising research hotspots, but the research and development of targeted drugs and the screening of appropriate clinical patients are clinical difficulties.

#### 4.7.4 Other treatments

Immunotherapy and hormone therapy may become potential novel options for salivary gland LEC in clinical settings.

#### 4.7.5 Prognosis

The prognosis of lymphoepithelial carcinoma varies widely across previous studies, with 5-year survival ranging from 50% to 90%. Local recurrence occurs in up to 28.9% of patients, while roughly 20% suffer distant metastasis within 3 years, predominantly to the lung, liver and brain. Cumulative survival rates for salivary gland lymphoepithelial carcinoma at 5, 10 and 15 years are 90%, 75% and 54% (38–40).In our study,case 1 and case 2 have been followed up for 15 months, with no local recurrence or distant metastasis identified during the follow-up period. The follow-up duration of case 3 is 21 months,hepatic metastasis was diagnosed at the 8th month after postoperative radiotherapy, and received surgery for the liver lesion.

## 5 Conclusion

LEC remains a rare salivary gland disease, so it is crucial not only to understand its epidemiology, pathogenesis, immunohistochemistry, pathological features, imaging and clinical manifestations, but also to correctly diagnose and differentiate LEC from other diseases. At present, combined treatment based on surgery and chemoradiotherapy for LEC of salivary gland is well-accepted, which aims to improve the prognosis of patients and improve their quality of life. With more in-depth molecular research, clinical experience accumulation, as well as the updated clinical drug research and development in the future, more scientific and effective diagnosis and treatment are expected among LEC of salivary gland in clinical settings.

## Data Availability

The original contributions presented in the study are included in the article/supplementary material. Further inquiries can be directed to the corresponding author.

## References

[1] Schneider M Rizzardi C . Lymphoepithelial carcinoma of the parotid glands and its relationship with benign lymphoepithelial lesions. Arch Pathol Lab Med. (2008) 132:278–82. doi: 10.5858/2008-132-278-lcotpg 18251590

[2] Hilderman WC Gordon JS Large HL Jr . Malignant lymphoepithelial lesion with carcinomatous component apparently arising in parotid gland. A Malignant counterpart of benign lymphoepithelial lesion? Cancer. (1962) 15:606–10. doi: 10.1002/1097-0142(196205/06)15:3<606::aid-cncr2820150322>3.0.co;2-u 13907296

[3] Redondo C Garcia A Vazquez F . Malignant lymphoepithelial lesion of the parotid gland: poorly differentiated squamous cell carcinoma with lymphoid stroma. Cancer. (1981) 48:289–92. doi: 10.1002/1097-0142(19810715)48:2<289::aid-cncr2820480213>3.0.co;2-9 7237400

[4] Saku T Cheng J Jen KY . Epstein-Barr virus infected lymphoepithelial carcinomas of the salivary gland in the Russia-Asia area: a clinicopathologic study of 160 cases. Arkhiv patologii. (2003) 65:35–9. 15357246

[5] Urano M Nakaguro M . The differential diagnosis of lymphoepithelial lesion of the salivary gland. Semin Diagn Pathol. (2024) 41:190–6. doi: 10.1053/j.semdp.2024.04.004 38734484

[6] Ban X Wu J Mo Y . Lymphoepithelial carcinoma of the salivary gland: morphologic patterns and imaging features on CT and MRI. AJNR Am J Neuroradiol. (2014) 35:1813–9. doi: 10.3174/ajnr.a3940 24831594 PMC7966265

[7] Zhang M Ye J Li H . Imaging features of lymphoepithelial like carcinoma of the salivary glands. Ear Nose Throat J. (2025) 104:NP426–31. doi: 10.1016/b978-0-443-36360-3.00032-8 35848422

[8] Yan F Lao WP Nguyen SA . Elective neck dissection in salivary gland Malignancies: Systematic review and meta analysis. Head Neck. (2022) 44:505–17. doi: 10.1002/hed.26923 34862810

[9] Zhan KY Nicolli EA Khaja SF . Lymphoepithelial carcinoma of the major salivary glands: Predictors of survival in a non-endemic region. Oral Oncol. (2016) 52:24–9. doi: 10.1016/j.oraloncology.2015.10.019 26547125

[10] Whaley RD Carlos R Bishop JA . Lymphoepithelial carcinoma of salivary gland EBV-association in endemic versus non-endemic patients: a report of 16 cases. Head Neck Pathol. (2020) 14:1001–12. doi: 10.1007/s12105-020-01172-w 32462279 PMC7669917

[11] Mozaffari HR Ramezani M Janbakhsh A . Malignant salivary gland tumors and Epstein-Barr virus (EBV) infection: a systematic review and meta-analysis. Asian Pacific J Cancer Prevention: APJCP. (2017) 18:1201. 10.22034/APJCP.2017.18.5.1201 PMC5555523 28610402

[12] He Q Zhou Y Fu C . Lymphoepithelioma is a nonkeratinizing squamous cell carcinoma with Epstein–Barr virus infection in China. J Cancer Res Ther. (2017) 13:807–12. doi: 10.4103/jcrt.jcrt_280_17 29237908

[13] Hipp JA Jing X Zarka MA . Cytomorphologic characteristics and differential diagnoses of lymphoepithelial carcinoma of the parotid. J Am Soc Cytopathology. (2016) 5:93–9. doi: 10.1016/j.jasc.2015.09.216 31042496

[14] Harada H Matsumoto H Nakatsuka S . Lymphoepithelial carcinoma of the parotid gland: a unique example showing p16 immunoreactivity. Med Mol Morphol. (2021) 54:368–73. doi: 10.1007/s00795-021-00295-5 34091759

[15] Picon H Guddati AK . Analysis of trends in mortality in patients with lymphoepithelial carcinoma of the head and neck. Int J Gen Med. (2021), 6245–50. doi: 10.2147/ijgm.s299145 34616177 PMC8488143

[16] Wong J Gologan O Ahmad K . Epstein–Barr virus-associated lymphoepithelial carcinoma arising in a salivary sebaceous lymphadenoma. Head Neck Pathol. (2023) 17:871–6. doi: 10.1007/s12105-023-01546-w 37022512 PMC10513992

[17] Adili A Oconnor T Wales P . Challenging tumor heterogeneity with HER2, p16 and somatostatin receptor 2 expression in a case of ebv-associated lymphoepithelial carcinoma of the salivary gland. Head Neck Pathol. (2023) 17:1052–7. doi: 10.1007/s12105-023-01592-4 37847488 PMC10739679

[18] Mong LC Liu KF Lin YH . Lymphoepithelial carcinoma in the sublingual gland. Int J Oral Maxillofac Surg. (2022) 51:869–73. doi: 10.1016/j.ijom.2021.08.024 34535351

[19] Wang CP Chang YL Ko JY . Lymphoepithelial carcinoma versus large cell undifferentiated carcinoma of the major salivary glands. Cancer. (2004) 101:2020–7. doi: 10.1002/cncr.20614 15389474

[20] Chowdhury Z Raphael V Khonglah Y . Mélange of lymphoepithelial lesions of salivary glands from a tertiary care center of North East India: Diagnostic conundrums. J Lab Physicians. (2021) 13:338–45. doi: 10.1055/s-0041-1731973 34975253 PMC8714313

[21] Shebl FM Bhatia K Engels EA . Salivary gland and nasopharyngeal cancers in individuals with acquired immunodeficiency syndrome in United States. Int J Cancer. (2010) 126:2503–8. doi: 10.1002/ijc.24930 19810095 PMC2847048

[22] Wenig BM . (2015). “ Lymphoepithelial-like carcinomas of the head and neck”, in: Seminars in diagnostic pathology ( WB Saunders), 32, 74–86. 10.1053/j.semdp.2014.12.004 25804344

[23] Thompson LDR Whaley RD . Lymphoepithelial carcinoma of salivary glands. Surg Pathol Clinics. (2021) 14:75–96. doi: 10.1016/j.path.2020.09.009 33526225

[24] Saw D Lau WH Ho JHC . Malignant lymphoepithelial lesion of the salivary gland. Hum Pathol. (1986) 17:914–23. doi: 10.1016/s0046-8177(86)80641-4 3759075

[25] Chou CT Ou CY Lee WT . Clinical features in salivary gland lymphoepithelial carcinoma in 10 patients: Case series and literature review. Laryngoscope Invest Otolaryngol. (2022) 7:779–84. doi: 10.1002/lio2.811 35734066 PMC9194977

[26] Punjabi LS Daryl Seow MK Ahmed SS . Lymphoepithelial carcinoma of the salivary gland—a great cytologic mimicker in the head and neck region, and the first report of SSTR2 expression on cytologic material. Diagn Cytopathol. (2022) 50:525–8. doi: 10.1002/dc.25038 35962726

[27] Deng D Zhou Q Ye Z . Clinical analysis of 12 patients with primary lymphoepithelial carcinoma of the parotid gland. Eur Arch Oto-Rhino-Laryngology. (2022) 279:2003–8. doi: 10.1007/s00405-021-06947-7 34379180

[28] Zhang G Tang J Pan Y . CT features and pathologic characteristics of lymphoepithelial carcinoma of salivary glands. Int J Clin Exp Path. (2014) 7:1004. doi: 10.1016/b978-0-443-36360-3.00032-8 24696717 PMC3971303

[29] Wang P Yang J Yu Q . Lymphoepithelial carcinoma of salivary glands: CT and MR imaging findings. Dentomaxillofacial Radiol. (2017) 46:20170053. doi: 10.1259/dmfr.20170053 28707954 PMC5965942

[30] Seok JY Lee KG . Cytologic features of metastatic lymphoepithelial carcinoma in pleural fluid: a case report. Acta Cytologica. (2009) 53:215–8. doi: 10.1159/000325128 19365979

[31] Pfister DG Spencer S Adelstein D . Head and neck cancers, version 2.2020, NCCN clinical practice guidelines in oncology. J Natl Compr Cancer Network. (2020) 18:873–98. doi: 10.6004/jnccn.2020.0031 32634781

[32] Qiu Z Wu Z Zhou X . Could definitive radiotherapy be a treatment option for lymphoepithelial carcinoma of major salivary gland: Comparison of clinical outcomes of upfront surgery and upfront chemoradiotherapy. Oral Oncol. (2023) 143:106443. doi: 10.1016/j.oraloncology.2023.106443 37295063

[33] Lv S Xie D Wu Z . Is surgery an inevitable treatment for advanced salivary lymphoepithelial carcinoma? Three case reports. Ear Nose Throat J. (2021) 100:NP402–6. doi: 10.1177/0145561320923170 32380853

[34] Niu X Liu P Zhou X . Is postoperative radiotherapy an essential treatment for nonmetastatic lymphoepithelial carcinoma of the salivary gland? Radiotherapy Oncol. (2022) 172:76–82. doi: 10.1016/j.radonc.2022.05.008 35568285

[35] Niu X Liu P Wang X . Is radical radiotherapy with/without surgery an effective treatment in the lymphoepithelial carcinoma of the salivary gland? BMC Cancer. (2023) 23:968. doi: 10.1186/s12885-023-11466-1 37828474 PMC10568878

[36] Kallel S Ayadi S Salem N . Lymphoepithelial carcinoma of the parotid gland. SAGE Open Med Case Rep. (2024) 12:2050313X241260210. doi: 10.1177/2050313x241260210 38868663 PMC11168047

[37] Wang X Luo Y Li M . Management of salivary gland carcinomas-a review. Oncotarget. (2017) 8:3946. doi: 10.1007/978-3-030-24059-2_14 27992367 PMC5354805

[38] Hsia B Tade Y Rafie S . Prognostic landscape of lymphoepithelial carcinoma: Analysis of a National Database. Cureus. (2025) 17:e78828. doi: 10.7759/cureus.78828 40084324 PMC11903371

[39] Wei J Deng H Wu L . Lymphoepithelial carcinoma of the head and neck: a SEER analysis of prognostic factors for survival. J Int Med Res. (2023) 51:3000605221148895. doi: 10.1177/03000605221148895 36650910 PMC9869209

[40] Fan Y Li C Qin J Lu H . Primary pulmonary lymphoepithelioma-like carcinoma. Med Oncol. (2020) 37:20. doi: 10.1007/s12032-020-1344-3 32146584

